# Factors Influencing Prone Positioning in Treating Acute Respiratory Distress Syndrome and the Effect on Mortality Rate

**DOI:** 10.7759/cureus.10767

**Published:** 2020-10-02

**Authors:** Ahmed Dardeir, Suganya Marudhai, Mauli Patel, Mohammad R Ghani, Vishal Busa

**Affiliations:** 1 Physical Medicine and Rehabilitation, California Institute of Behavioral Neurosciences and Psychology, Fairfield, USA; 2 Internal Medicine, California Institute of Behavioral Neurosciences and Psychology, Fairfield, USA; 3 Neurology, California Institute of Behavioral Neurosciences and Psychology, Fairfield, USA

**Keywords:** prone positioning, acute respiratory distress syndrome, ards

## Abstract

Acute respiratory distress syndrome (ARDS) is often associated with severe hypoxemia and a high mortality rate. Prone positioning is a well-established intervention for ARDS. It has been shown to improve oxygenation and prevent ventilator-induced lung injury due to the more uniform distribution of lung stress and strain. This narrative review aims to compare the various factors that may influence how prone positioning affects mortality rates. We will examine the duration of time a patient is in the prone position, severity of ARDS, use of lung-protective ventilation, and the time elapsed between ARDS diagnosis and placing a patient in the prone position. A literature review on prone positioning in ARDS was performed and searched data from PubMed and Google Scholar for articles published from 2010 to 2020. Although no single variable used during prone positioning reduces mortality rates in ARDS patients, combining several optimal conditions may yield increased survival benefits. Early initiation of extended prone positioning sessions combined with low tidal volumes shows encouraging results in severe ARDS patients. Future research on this subject should focus on further examining these variables in a study enrolling a larger number of subjects in a setting with adequately trained staff familiar with proper prone positioning techniques.

## Introduction and background

Acute respiratory failure (ARF) is a potentially fatal illness occurring when gas exchange in the lungs is impaired [1]. The two types of respiratory failure - hypoxic and hypercapnic - are distinct in that one is associated with hypoxia (type 1), and the other is a ventilation disorder (type 2) [1]. ARF is a common occurrence in the intensive care unit (ICU). More than 75% of patients require mechanical ventilation during their ICU stay, resulting in fatality for 33% to 37% of mechanically ventilated patients [1]. Acute respiratory distress syndrome (ARDS) is a form of hypoxemic respiratory failure resulting from acute inflammatory lung injury [2]. Patients treated for ARDS typically have prolonged hospitalizations with resultant increased hospital costs and overall greater consumption of resources [2,3]. Those patients who survive are often burdened with disabilities such as muscle weakness and neuropsychiatric problems for months and even for years after being discharged from the hospital [4].

A patient in ICU who requires mechanical ventilation is typically attached to numerous lines and tubes and is not often turned from the supine position [1]. Also, nursing care is more conveniently provided when the patient is supine [1,5]. Immobility has significant effects on the lungs, resulting in atelectasis and pneumonia [1,6]. Rotating the patient into the prone position was first proposed in 1974 [6]. Some noted benefits of prone positioning were improved oxygenation and even reduced mortality rates [5-7]. Physiological effects include improved oxygenation, decreased atelectasis, an increase in the average ratio of PaO_2_/FiO_2_ by 35 mm Hg, and a reduction in the relative shunt fraction by approximately 30% [1,2,8]. Placing a patient in a prone position may also lessen the potential complications of ventilator-associated infections such as pneumonia by promoting drainage of secretions from the dorsal lung to the ventral trachea [5,7,8]. The correlation between prone positioning and high positive end-expiratory pressure (PEEP) ventilation has been explored, and evidence suggests prone positioning may reduce the adverse effects of PEEP, decreasing barotrauma and subsequent ventilator-induced lung injury [8]. Overall improved oxygenation during prone positioning has reparative benefits to the lungs and reduces secondary lung infection/injury, resulting in decreased mortality rates [5].

While prone positioning is mentioned in the literature as beneficial for the reasons noted above, several studies have reviewed the variables that can affect recovery and mortality. Such variables that have been investigated are the timing of treatment, duration of time a patient was in the prone position, tidal volume relative to body weight, and severity of ARDS [2,5,9-11].

This narrative review aims to review the effect of prone positioning in patients with ARDS on mortality rate. More specifically, we will look at variables such as duration and initiation of the prone position, use of lung-protective ventilation, the severity of ARDS, and how they affect recovery.

Methodology

A literature search of PubMed and Google Scholar databases was conducted using the following MeSH term and keyword combination: "prone positioning" AND "acute respiratory distress syndrome (ARDS)” which identified 575 studies. We included studies published from 2010 to 2020 which produced 275 studies. The search was further narrowed to identify studies related to prone positioning in patients with ARDS and was limited to English language studies involving human subjects which identified 238 studies. All article types were included. The final reference list was determined based on relevance to the topics discussed in this review. 

## Review

Duration of prone positioning

An issue physicians often face when initiating prone positioning as an intervention is an optimal duration the patient should remain prone [12,13]. Duration has been examined as a possible variable affecting mortality in several published studies [13]. At one time, a six-hour window was selected because it coincided with the typical nursing shift. Prior to 2005, most published studies utilized shorter prone duration, whereas, after 2005, researchers implemented a longer duration of prone positioning [14]. It is worth noting that a longer duration in the later studies often coincided with lower tidal volume mechanical ventilation, which may affect mortality. Gattinoni et al. looked at 304 patients placed in prone for an average of seven hours per day; however, they found no benefit on survival or mechanical ventilation duration during the ICU course despite noting improvements in oxygenation [15]. Voggenreiter et al. conducted a randomized trial of 40 subjects placed in the prone position for 11 (±5) hours [16]. They concluded that although the duration of mechanical ventilation was not reduced, patients experienced improvements in oxygenation as well as a reduction in the prevalence of ARDS and pneumonia. The sample size of the study was too small to determine the effects on mortality. Mancebo et al. utilized some of the more progressive theories developing in the literature [17]. They employed a much longer duration of prone positioning (17 hours/day) with the 136 subjects enrolled in their study (58% ventilated in the supine position and 43% ventilated in prone position). Results indicated a 15% absolute and 25% relative reduction in ICU mortality compared to patients who remained in the supine position, although these results did not achieve statistical significance. Taccone et al. examined the effects of the prone position on 168 subjects prone for 18 hours per day [18]. Their findings indicated a nonsignificant 15% reduction in mortality in the prone group. In a well-known study known as the proning severe ARDS patients (PROSEVA) trial, Guerin et al. 2013 randomly assigned 466 patients to either a prone group (237 patients) or a supine group (229 patients) [10]. Patients remained in a prone position for at least 17 hours per day [10]. They concluded that early initiation of the prone position in severe ARDS cases significantly reduced 28-day mortality (16% in the prone group and 32.8% in the supine group) and 90-day mortality (23.6% in the prone group and 41% in the supine group) [10].

When comparing the studies mentioned earlier, no reduction in mortality rate was noted by Taccone et al. [17] and Mancebo et al. [18], both of which employed longer duration of prone positioning per day (18 hours/day and 17 hours/day, respectively). Gattinoni et al. [15] and Voggenreiter et al. [16] also found no significant impact on survival when patients were placed in the prone position. However, other benefits of prone positioning are cited in these studies. Guerin et al. was the only study to achieve a statistically significant reduction in mortality rate [10]. Although they all employed a similar duration of prone positioning, other variables were not consistent across the studies. These included sample size (Taccone - 168 subjects, Mancebo - 136 subjects, and Guerin - 466) and tidal volumes employed during mechanical ventilation [17,18]. It appears reasonable to surmise that duration may be a necessary factor when initiating a prone positioning protocol in an ICU setting. 

Initiation of prone positioning

It is beneficial to examine the effects of initiating the prone position early in the ICU course to prevent lung injury versus later when lung injury may be more advanced and possibly irreversible. Fernandez et al. noted that prone positioning is less effective when initiated later in the ICU course as a "rescue therapy" when a patient experiences refractory ARDS [19]. There are several reasons a physician may not initiate prone positioning until it is necessary as a "rescue therapy," such as fear of complications, lack of staff familiarity with the proper prone positioning technique, the resistance of staff, increased sedation needs, and changes in enteral feeding schedules [14,20]. Mancebo et al. enrolled patients within 48 hours of ARDS diagnosis [17]. They found that the number of days elapsed between ARDS onset and study entry was an independent risk factor for mortality. Fernandez et al. enrolled 40 patients (19 supine and 21 prone) in their study within 48 hours of ARDS diagnosis [19]. Results indicated a 15% increase in survival rate; however, this outcome did not achieve statistical significance due to the small sample size [19]. Guerin et al. enrolled 466 patients in the study 12-24 hours after ARDS was diagnosed [10]. It should be noted that they enrolled patients with more severe ARDS cases, as defined by levels of PaO_2_/FiO_2_ < 150 mm Hg. They found a significant reduction in mortality at 28 and 90 days as follows: 16% in the prone group/32% in the supine group at 28 days and 23.6% mortality in the prone group/41% mortality in the supine group at 90 days.

All three studies reviewed above recognized some benefit to placing patients in the prone position within 48 hours of ARDS diagnosis. However, the results achieved statistical significance only in the study by Guerin et al. [10]. Although it cannot be determined whether initiation of prone positioning alone impacted the results, timing should be considered a necessary element of developing a prone positioning protocol [1-2,5,7-8]. Unless a patient has specific risk factors for which prone positioning is contraindicated, it seems plausible to initiate this intervention as early as possible, considering there are clear benefits to initiating the prone position, i.e., improved oxygenation, decreased atelectasis, and decreased risk of ventilator-associated pneumonia.

Lung-protective ventilation

A prevalent concern for physicians when a patient is mechanically ventilated is the risk of ventilator-induced lung injury, which can often be prevented by lung-protective ventilation [21,22]. Lung-protective ventilation, defined as 6 ml/kg of predicted body weight, has specifically been shown to improve overall outcomes and decrease the risk of ventilator-induced lung injury in ARDS patients [23]. Of the several prominent studies that examined the effects of prone positioning in ARDS, not all included lung-protective ventilation, as they employed tidal volumes ≥10 ml/kg of predicted body weight [15,17]. The ARDS Network officially instituted a protocol for ARDS treatment, which instructs tidal volumes to be set initially no higher than 8 ml/kg and decreased by 1 ml/kg at intervals of ≤2 hours until a goal rate of 6 ml/kg is achieved [22]. Subjects included by Gattinoni et al. and in the 2004 study by Guerin et al. did not have the benefit of lung-protective ventilation since tidal volumes were set at ≥ 10 ml/kg, whereas Mancebo et al. and Taccone et al. employed the use of lung-protective ventilation with tidal volumes <10 mL/kg [15,17,18,24]. In the 2013 study by Guerin et al., strict lung-protective ventilation guidelines were followed with tidal volumes set at 6 mL/kg [10].

In the initial two studies by Gattinoni et al. and Guerin et al., no survival benefit was realized [15,24]. In the study by Mancebo et al. and Taccone et al., a reduction in ICU mortality was noted; however, this outcome did not achieve statistical significance [17,18]. In the 2013 study by Guerin et al., a significant reduction in mortality was noted, which they attribute to, among other factors, reduced ventilator-induced lung injury [10]. When comparing tidal volumes used across all five studies, it seems the only study that achieved a statistically significant reduction in mortality was the 2013 study by Guerin et al. [24]. One variable that differentiates Guerin et al. from the other four studies is the use of low tidal volumes (6 mL/kg). Perhaps this is an essential factor in reducing ventilator-induced lung injury, and when combined with prone positioning, significant benefits are realized. 

Severity of ARDS

The efficacy of prone positioning as it relates to the severity of ARDS is a factor that is frequently addressed in the research. Severity is stratified as moderate (PaO_2_/FiO_2_ ≥ 100 mmHg) or severe (PaO_2_/FiO_2 _< 100 mmHg) [14]. It has been theorized that the beneficial effects of prone positioning are due to increased lung recruitment, and patients with severe hypoxia have more "recruitable lung" potential [25]. Gattinoni et al. indicated improved mortality in patients with the most severe hypoxemia (PaO_2_/FiO_2_ ratio ≤ 88) and a high Simplified Acute Physiology Score II (>49); however, this did not reach statistical significance [15]. Taccone et al. also subdivided their subjects into moderate (PaO_2_/FIO_2_ 100-200 mm Hg) and severe (PaO_2_/FIO_2_ < 100 mm Hg) ARDS to test the theory that prone positioning may only benefit the most severely ill subjects with ARDS [18]. Although their results did not demonstrate a significant survival benefit in either population, they detected a trend favoring the group with severe hypoxemia [18]. Lastly, Guerin et al. saw a 28- and 90-day mortality decrease in their study, which only enrolled patients with severe ARDS [10]. 

Only one of the three studies mentioned above realized significant survival benefits favoring moderate versus severe ARDS; however, all groups noted trends that support increased benefit for more severe cases [10,15,18]. Some researchers attribute the efficacy of treatment in any disease to the mortality rate of the control group [19]. Illnesses with high mortality, such as severe ARDS, are often more likely to demonstrate benefits. 

Though many studies have investigated the effects of prone position in ARDS, our review indicates that results have not always achieved statistical significance in favor of reducing mortality rates. One of the most well-known studies conducted by Guerin et al. in 2013 did report a significant survival benefit [10]. When considering all the variables that may have affected the outcomes across the various studies, Guerin et al. is a critical study to examine in more detail so healthcare professionals can begin implementing those variables into a standardized prone positioning protocol in the ICU. 

Guerin et al. employed the lowest tidal volumes of all the major studies (6 mL/kg), demonstrating their firm adherence to the use of lung-protective ventilation [10]. Prone positioning duration was 17 hours per day, which was not unique to their study. Similar times are noted in the studies by Mancebo et al. and Taccone et al. [17,18]. Another notable factor is the level of staff experience with prone positioning techniques. Guerin et al. only used centers with substantial experience combined with multimedia-based training focused on prone positioning [26]. Lastly, they limited their recruitment of severe ARDS subjects, defined as PaO_2_/FiO_2_ < 150 mm Hg [10]. 

Despite certain studies reporting some favorable benefits of prone positioning when used as an ARDS intervention, studies have indicated that it is underused among ICU physicians [27]. Issues that prevent implementing a prone positioning protocol in an ICU setting might include lack of training, staffing constraints, particularly during overnight shifts, physician or nurse discomfort, and shortage of equipment. Adverse effects of prone positioning have also been examined, including unintentional extubation, obstruction of the endotracheal tube, the formation of pressure sores, increases in ocular pressure, loss of venous access, increased gastric residuals, displacement of the catheter, need for increased sedation, and intracranial hypertension [4,5,8,9,13,28]. It is further noted that physicians will only consider prone positioning as an intervention for patients with low risk for complications versus high-risk patients, i.e., those with facial/neck trauma, elevated intracranial pressure, or patients at high risk for requiring CPR [8,26]. Lastly, the case-mix of ARDS severity in some ICU's may not indicate the need for prone positioning as often as ICU's that treat a higher number of ARDS patients [27].

**Figure 1:**
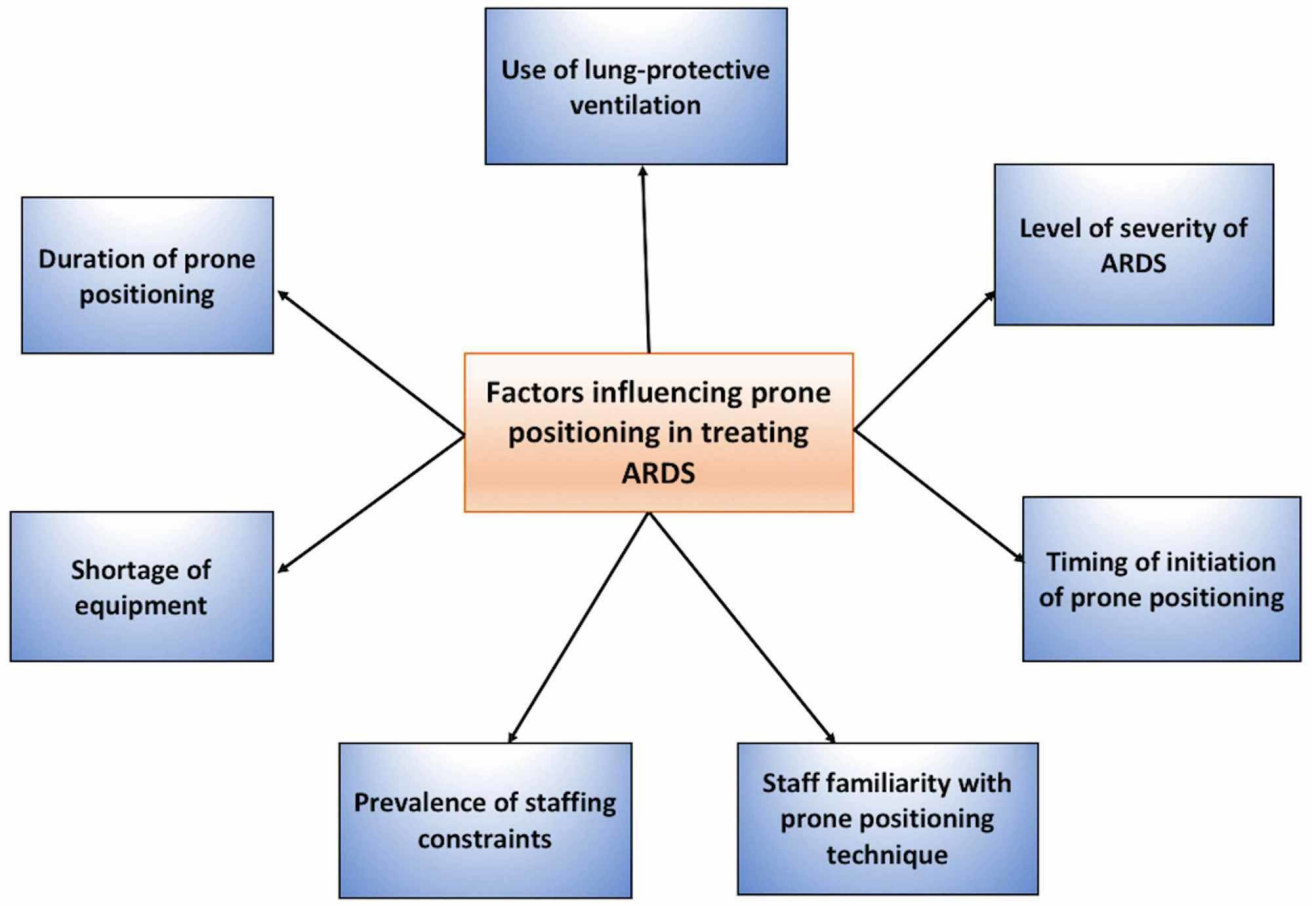
Various factors influencing prone positioning in treating ARDS ARDS: acute respiratory distress syndrome.

Prone positioning as a treatment for ARDS has potential; however, support in the research has been incongruous. Although it is documented as an intervention that will significantly impact the ICU patient's treatment course, several studies we reviewed did not demonstrate a survival benefit [6,17,18]. Since the patient in ICU is medically complex, several variables might exist in each case, such as length of time intubated, the severity of ARDS, the use of lung-protective ventilation, timing at which prone positioning was initiated, use of neuromuscular blocking agents, and tidal volumes [6]. Figure 1 summarizes some of these and other previously discussed variables that influence the effectiveness of prone positioning in the treatment of ARDS. It is essential to identify the standards of care that physicians in ICU can adopt when developing prone positioning protocols for ARDS treatment.

Limitations

The limitation of this narrative review is that there is no single factor that can increase the effectiveness of prone positioning in ARDS patients. Also, there were no quality assessments to standardize prone positioning techniques used across the studies as well as no specified patient populations (i.e., ethnicity, age, and severity of the disease).

Future directions

Future studies should employ all the relevant variables mentioned in this review and include a larger pool of subjects. Studies should also systematically control the level of staff training, as adequate training will likely account for more successful outcomes. 

## Conclusions

In this review, we sought to examine the current literature to determine how prone positioning's effectiveness is influenced by variables such as duration, time of initiation, use of lung-protective ventilation, and ARDS severity. The majority of studies found no statistically significant impact on mortality in the prone position group compared with their control group. It appears that no single factor can increase the effectiveness of prone positioning; however, when several optimal variables are integrated along with stringent staff education, a survival benefit is more plausible. The literature indicates that early initiation of extended prone positioning sessions is favorable. Adherence to lung-protective ventilation during prone position is also an important factor in preventing ventilator-induced lung injury, which may correlate with a reduction in mortality. Lastly, severe ARDS subjects often experienced increased benefit from prone positioning compared to moderate cases. Future research that enrolls a larger number of subjects and employs all the factors mentioned above would be beneficial, allowing physicians to take a more standardized approach to develop a prone positioning protocol.
